# Prolonged operative time of repeat cesarean is a risk marker for post-operative maternal complications

**DOI:** 10.1186/s12884-018-2111-8

**Published:** 2018-12-04

**Authors:** Misgav Rottenstreich, Hen Y. Sela, Ori Shen, Rachel Michaelson-Cohen, Arnon Samueloff, Orna Reichman

**Affiliations:** Department of Obstetrics and Gynecology, Shaare Zedek Medical Center, Hebrew University, Jerusalem, Israel

**Keywords:** Repeat caesarean deliveries, Post-operative adverse maternal outcome, Operative time

## Abstract

**Background:**

Repeat cesarean delivery (CD) accounts for approximately 15% of all annual deliveries in the US with an estimated 656,250 operations per year. We aimed to study whether prolonged operative time (OT; skin incision to closure) is a risk marker for post-operative maternal complications among women undergoing repeat CD.

**Methods:**

We conducted a cross-sectional retrospective study in a single tertiary center including all women who underwent repeat CD but excluding those with cesarean hysterectomy. Prolonged OT was defined as duration of CD longer than the 90th percentile duration on record for each specific surgeon in order to correct for technique differences between surgeons. Bi-variate analysis was used to study the association of prolonged OT with each one of the following maternal complications: post-operative blood transfusion, prolonged maternal hospitalization (defined as hospitalization duration longer than 1 week post-CD), infection necessitating antibiotics, re-laparotomy within 7 days post-CD, and re-admission within 42 days post-CD. A multivariate regression analysis was performed controlling for maternal age, ethnicity, parity, number of fetus, gestational age at delivery, trial of labor after cesarean, anesthesia, and number of previous CDs. The adjusted odd ratio was calculated for each complication independently and for a composite adverse maternal outcome defined as any one of the above.

**Results:**

A total of 6507 repeat CDs were included; prolonged OT was highly associated (*P* value < 0.000) with: post-operative blood transfusion (4.4% vs. 1.5%), prolonged hospitalization (8.4% vs. 4.0%), infection necessitating antibiotics (2% vs. 1%), and readmission (1.8% vs. 0.8%) when compared to control. The composite adverse maternal outcome was also associated with prolonged OT (20.2% vs. 11.2%, *p* < 0.000). These correlations remained statistically significant in the multivariate regression analysis when controlling for confounders.

**Conclusions:**

Among women undergoing repeat CD, prolonged OT (reflecting CD duration greater than 90th percentile for the specific surgeon) is a risk marker for post-operative maternal complications.

## Background

During the last three decades, the decrease in trial of labor after cesarean delivery (TOLAC), has been reflected in an inversely increased fraction of repeat cesarean deliveries (CD) [1, 2].

It is currently estimated that one-third of all CD in the United States are repeated CD and more than 90% of women who undergo CD will have a repeat procedure in subsequent pregnancies [3, 4].

Large observational studies have consistently shown that women who undergo repeat CD are at increased risk of dense adhesions and abnormal placentation [5–10]. These in turn make the surgical procedure and fetal extraction more challenging, prolong the time to delivery, and increase the risk of bowel or bladder injury [11–13] Both dense adhesions and abnormal placentation are associated with massive hemorrhage, disseminated intravascular coagulation, blood products transfusion, admission to intensive care unit (ICU), and in severe cases could result in maternal death [10].

A few studies have been published analyzing the association between CD operative time (OT) and maternal or neonatal complications [11, 14]. However, these studies combined primary and repeat CD and disregarded identification of the primary surgeon, a factor that can potentially influence OT due to differences in techniques between surgeons [15]. We aimed to study OT based on the individual surgeon’s historical record of OT and post-operative maternal complications among women undergoing repeat CD. We defined prolonged OT as duration of CD surgery longer than the 90th percentile of that specific surgeon’s recorded CD durations. Our primary aim was to ascertain whether there was a correlation between prolonged OT and (1) hemorrhage necessitating post-operative blood transfusion; (2) length of maternal hospitalization (3); infection necessitating administration of antibiotics (4); re-laparotomy within 7 days of CD; and (5) re-admission within 42 days of discharge.

## Materials and methods

A retrospective cross sectional study was conducted at Shaare Zedek Medical Centre (SZMC), a university affiliated medical center, managing approximately 10% of all deliveries in Israel, with an average of 13,000 deliveries annually. National Health plans cover antenatal and peripartum care, and most deliveries (> 95%) are managed by this system. The study population included all women who underwent a repeat CD by an obstetrician who had performed at least ≥40 CD at SZMC between June 2005 and December 2014. Since hysterectomies occur in 0.6 cases/1000 deliveries and invariably prolong OT as well as being associated with higher maternal morbidity, we excluded all cases of hysterectomies regardless of indication.

The SZMC obstetrical database is continuously updated from the computerized electronic medical records (EMR)which are updated in real-time during labor and delivery. The EMR includes demographic characteristics, medical and obstetric history, and requisite descriptive variables as per the computerized surgical notes, such as inter-pregnancy interval, number of previous CD, elective CD versus TOLAC, type of anesthesia, OT (skin incision to closure), experience of the operating surgeon and presence of a third surgeon. Complications that were studied included (1) clinically significant hemorrhage defined as post-operative blood transfusion; (2) prolonged length of maternal hospitalization defined as length of stay following CD greater than 7 days (departmental protocol mandates discharge on day 5 post-operation); (3) infection necessitating antibiotics [including: Gentamycin, Amoxicillin/Clavulanate potassium, Ciprofloxacin and Cefazolin as markers for endometritis, wound infection, post-operative intra-abdominal infected collections and urinary tract infections]; (4) re-laparotomy within 7 days of the CD; and (5) re-admission within 42 days of discharge.

Prolonged CD was defined as surgery where the OT was longer than the 90th percentile of CD durations on record for that specific surgeon. According to department policy an attending must be present at all CD; OT is attributed to the primary surgeon regardless of seniority. Bivariate analysis was performed for testing the association between prolonged CD and categorical variables using the *χ*^2^ or Fisher exact test and continuous variables were analyzed by the Student’s t-test as indicated. A multivariate regression analysis was performed controlling for confounders, and adjusted odds ratio were calculated. The sensitivity, specificity, positive and negative predictive value of prolonged OT for each complication and for the composite adverse outcome were calculated. Confidence interval of 95% and *P* value ≤0.05 were defined as statistically significant. All statistics were performed using the SPSS version 23. The study was approved by the SZMC IRB (# 0260–16).

## Results

**Fig. 1:**
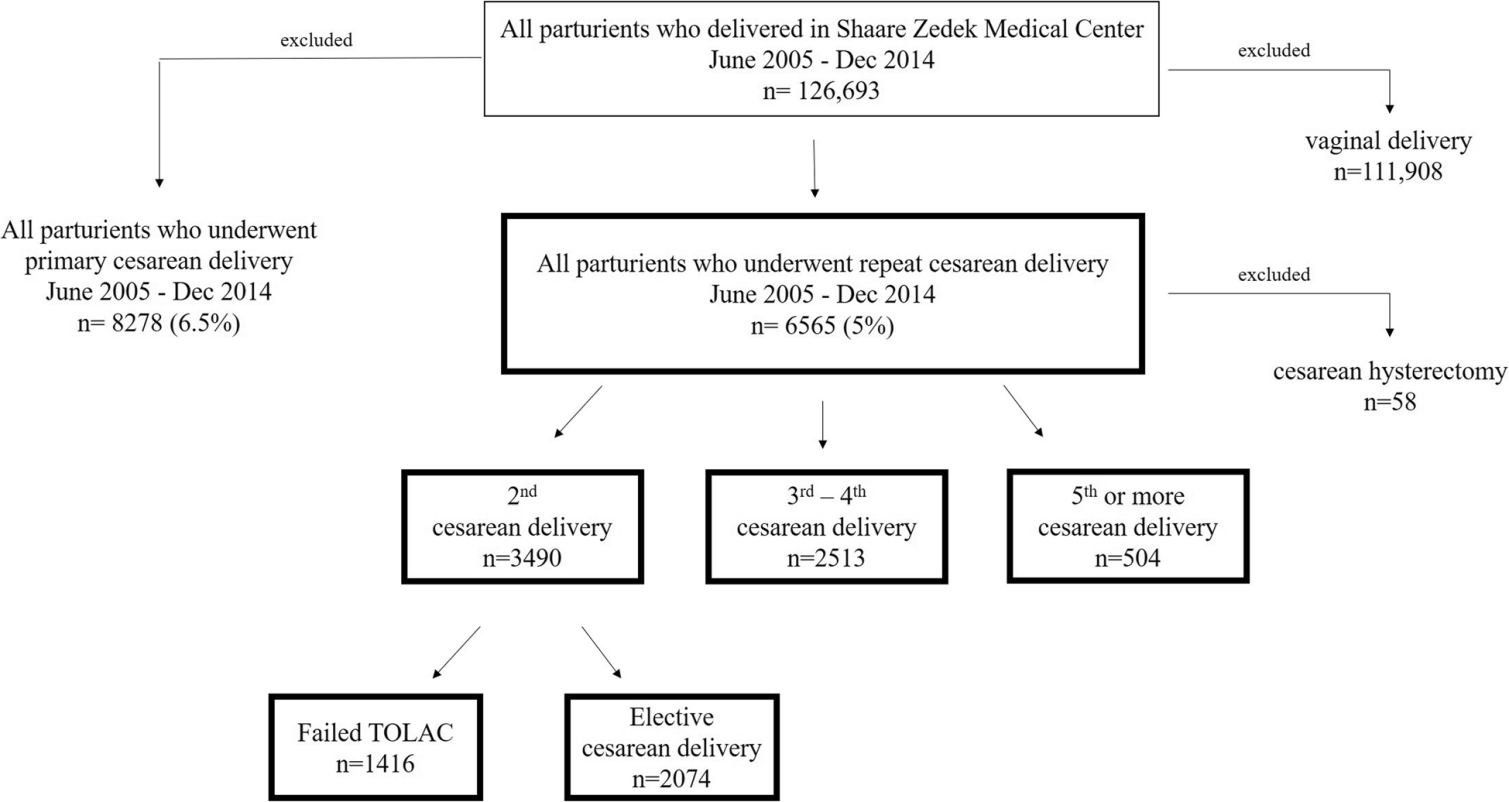
flow chart of study population

During the study period, 14,875 CD were performed out of 126,693 total deliveries (11.6%). Of the CD 6507 (43.7%) were repeat CD. Approximately one-fifth of the study population, 1416 (22%) were repeat CD due to failed TOLAC (Fig. 1- flow chart). Fifty-nine obstetricians were included in the analysis: 23 board certified obstetrics and gynecology and 36 residents, with comparable numbers of CD (median of 200 and 190. respectively). Mean duration of CD ranged between surgeons with a minimum of 30 (±13) minutes to a maximum of 66 (±30) minutes. Demographic characteristics, medical and obstetric history, and clinical features of the study population stratified by OT are presented in Table 1. One-fifth of the study population 1293 (20%) was grand-multiparous (≥6 deliveries). Prolonged CD duration relative to expected CD duration per surgeon’s history was highly associated (*P* value < 0.000) with: gestational age at delivery, general anesthesia, number of previous CDs, TOLAC; and presence of a third surgeon (Table 1). There was a strong association between prolonged CD and post-operative adverse maternal outcomes including post-operative blood transfusion (4.4% vs. 1.5%), prolonged hospitalization (8.4% vs. 4.0%), infection necessitating antibiotic treatment (2% vs. 1%) and readmission (1.8% vs 0.8%) (Table 2). These significant correlations remained in multivariate regression analysis controlling for confounders (Table 2). The sensitivity, specificity, positive and negative predictive values of prolonged OT for each of the post-operative maternal complications and for the combined composite complication is presented in Table 3. There was a high negative predictive value (NPV > 0.93) for all complications studied.

**Table 1:** Sociodemographic and obstetrical characteristic of study population, distributed by length of surgery

		Length of cesarean surgery^a^		
		≤ 90th percentile	> 90thpercentile	*P* value
Age		33.0 ± 5.4	33.6 ± 5.3	< 0.000
Education	< 12 years	217 (4.1%)	52 (5.0%)	0.188
Ethnicity	Jew	4721 (86.7%)	894 (84.0%)	0.019
	Arab	722 (13.3%)	170 (16.0%)	
Parity	Multipara 2–5^c^	4150 (76.2%)	772 (72.6%)	0.010
	GMP 6–9	1029 (18.9%)	232 (21.8%)	
	GGMP≥10	264 (4.9%)	60 (5.6%)	
Interval from previous CD (months)^b^	< 12 months	434 (16.3%)	89 (16.5%)	
	12–24	1027 (38.5%)	184 (34.1%)	0.139
	> 24 months^c^	1206 (45.2%)	266 (49.4%)	
Number offetus	singleton	5204 (95.6%)	1032 (97%)	0.039
	multiple pregnancy	239 (4.4%)	32 (3.0%)	
Gestational week	term	4738 (87.1%)	838 (78.8%)	< 0.000
	preterm < 37	704 (12.9%)	226 (21.2%)	
Anesthesia	local	5246 (96.5%)	958 (90%)	< 0.000
	general	188 (3.2%)	106 (10.0%)	
Cesarean delivery	elective^c^	3219 (59.1%)	548 (51.5%)	
	elective in labor	94 (1.7%)	20 (1.9%)	< 0.000
	emergent	2126 (39.1%)	496 (46.6%)	
Number of CD	2^c^	3023 (55.5%)	467 (43.9%)	< 0.000
	3–4	2040 (37.5%)	473 (44.5%)	
	≥5	380 (7.0%)	124 (11.7%)	
Experience of surgeon	senior > 10 yrs. ^c^	1709 (32.6%)	333(31.3%)	
	senior < 10 yrs.	430 (8.2%)	102 (9.6%)	0.289
	resident	3111 (59.3%)	629 (59.1%)	
Presence of a third surgeon in surgery	non-present	5443 (100%)	1052 (98.9%)	< 0.000
	present	0 (0%)	12 (1.1%)	
Fetal weight	macrosomia > 4000	354 (6.5%)	61 (5.7%)	
	AGA 2500–4000^c^	4468 (82.3%)	856 (80.5%)	
	LBW 1500–2500	523 (9.6%)	120 (11.3%)	0.059
	VLBW 1000–1500	60 (1.1%)	16 (1.5%)	
	ELBW < 1000	24 (0.4%)	10 (0.9%)	

*GMP* grand-multipara

^a^The 90th percentile was calculated individually for each surgeon

^b^missing data 3301 (50.7%)

^c^reference variable in the multivariate analysis

**Table 2:** Post-operative maternal complications distributed by length of surgery, univariate and multivariate analysis^a^

	Univariate analysis (chi square)			Multivariate analysis (Adjusted Odds Ratio)	
	> 90th percentile	> 90th percentile	*p* value	aOR	95% CI
Post-operative blood transfusion	82 (1.5%)	47 (4.4%)	< 0.000	3.16	2.17–4.61
Prolonged length of hospitalization (≥ 7 days)	216 (4%)	89 (8.4%)	< 0.000	1.89	1.44–2.46
Rx with antibiotics^b^	393 (7.2%)	128 (12%)	< 0.000	1.59	1.28–1.98
Re- admission	42 (0.8%)	18 (1.8%)	0.004	1.94	1.10–3.44
Composite complication^c^	607 (11.2%)	215 (20.2%)	< 0.000	1.81	1.52–2.16

^a^multivariate analysis controlling for maternal age, ethnicity, parity, number of fetus, gestational age at delivery, urge of operation, anesthesia and number of cesarean delivery

^b^Rx with either Gentamycin, Augmentin, Ciprofloxacin, Cefazolin

^c^combining post-operative blood transfusion, prolonged length of hospitalization, treatment with antibiotics and readmission

**Table 3:** The sensitivity, specificity, positive and negative predictive value of prolonged cesarean delivery (> 90th percentile) to each one of the post-operative maternal complications

	sensitivity	specificity	PPV	NPV
Post-operative blood transfusion	0.36	0.85	0.15	0.99
Prolonged length of hospitalization^a^	0.29	0.84	0.08	0.96
Rx with antibiotics^b^	0.25	0.84	0.12	0.93
Re- admission	0.30	0.84	0.02	0.99
Composite post-operative complication^c^	0.26	0.85	0.20	0.89

*PPV* positive predictive value, *NPV* negative predictive value

^a^prolonged length of maternal hospitalization defined as discharge after 7 days or more

^b^Rx with either Gentamycin, Augmentin, Ciprofloxacin, Cefazolin

^c^combining post-operative blood transfusion, prolonged length of hospitalization, necessitating antibiotic treatment and readmission

## Discussion

This study confirms our hypothesis that prolonged CD defined as OT greater than the 90th percentile of CD durations on record for each surgeon, was associated with post-operative maternal complications including post-operative blood transfusion, prolonged maternal hospitalization, treatment with antibiotics and readmission. Therefore, prolonged CD can be seen as a **risk marker** for adverse maternal outcomes. Nonetheless, the low prevalence of these complications (Table 3), limits our predictive ability, i.e., which women with prolonged OT will actually suffer complications (positive predictive value of 0.02–0.20). On the other hand, OT within the surgeon’s (<90th percentile) duration, can reassure women with a high degree of confidence that they probably are not at risk of the above complications.

Moreover, the negative predictive value for each one of the complications studied (0.93–0.99) implies that for women undergoing repeat CD within the surgeon’s < 90th percentile duration, are less likely to suffer the adverse complications analyzed herein.

There are few studies regarding the effect of prolonged CD on maternal and fetal morbidity [11, 14]. These studies are limited as they grouped together primary and repeat CD and the later arbitrary defined prolonged operation as below and above 30 and 60 min [14]. As clinicians, we were concerned that defining OT as a fixed number without considering the surgeon who operated is problematic because of variability in techniques among surgeons regardless of seniority. In order to correct for the effect of the primary surgeon we defined prolonged CD as ≥90th percentile calculated individually for the primary surgeon. We attributed operative time to the primary surgeon who could have been a resident and not the attending physician. In addition, to minimize bias of confounders and modifications, we included only women undergoing repeat CD and not primary CDs that are by nature different than repeat CD [16].

There are some limitations inherent in this study. First, this is a retrospective, cross-sectional single center university hospital, yet we believe that for the research question addressed this methodology is equitable. Our study population with high parity may weaken external validity, yet it also enabled inclusion of a large number of parturients undergoing repeat CD: 2513 women underwent their 3rd or 4th CD and 504 parturient underwent their 5th CD or more.

Unfortunately body mass index which is an essential factor associated with maternal morbidity is not currently available in our computerized database such. Despite these limitations, the large study group along with the requisite variables that continuously updated in real time, decreases bias.

In conclusion, among women undergoing repeat CD, prolonged CD is a **risk marker** for post-operative maternal complications. In addition, OT < 90th percentile of the surgeon’s on record CD duration has high NPV for adverse maternal outcome, implying **who will not** suffer the studied complications. We encourage surgeons and institutions to obtain OT of CD and calculate the 90th percentile for each surgeon. This will enable the surgeon, in case of OT within the normal limits (< 90th percentile), to reassure the patient and their family immediately after the CD that the risk of post-operative complication including blood transfusion or prolonged admission are very low.

## Conclusions

Prolonged OT, beyond the surgeon’s 90th percentile of on record CD durations, is a risk marker for post-operative maternal complications among women undergoing repeat CD. In case of prolonged OT, the surgeon may elect for more careful follow-up of the patient based on the local standard protocol.

## Abbreviations

CD: Cesarean delivery
ICU: Intensive care unit
OT: Operative time
SZMC: Shaare Zedek Medical Centre
TOLAC: Trial of labor after cesarean delivery
